# Penetration of Triphenylphosphonium Derivatives through the Cell Envelope of Bacteria of *Mycobacteriales* Order

**DOI:** 10.3390/ph16050688

**Published:** 2023-05-02

**Authors:** Pavel A. Nazarov, Konstantin B. Majorov, Alexander S. Apt, Maxim V. Skulachev

**Affiliations:** 1 Belozersky Institute of Physico-Chemical Biology, Lomonosov Moscow State University, 119991 Moscow, Russia; max@mitotech.ru; 2 Central Research Institute for Tuberculosis, 107564 Moscow, Russia; majorov@list.ru (K.B.M.); alexapt0151@gmail.com (A.S.A.); 3 Mitotech LLC, 119991 Moscow, Russia

**Keywords:** bacterial cell envelope penetration, triphenyl phosphonium, phytopathogens, tuberculosis, mitochondria-targeted antioxidants, antibiotics

## Abstract

The penetration of substances through the bacterial cell envelope is a complex and underinvestigated process. Mitochondria-targeted antioxidant and antibiotic SkQ1 (10-(plastoquinonyl)decyltriphenylphosphonium) is an excellent model for studying the penetration of substances through the bacterial cell envelope. SkQ1 resistance in Gram-negative bacteria has been found to be dependent on the presence of the AcrAB-TolC pump, while Gram-positive bacteria do not have this pump but, instead, have a mycolic acid-containing cell wall that is a tough barrier against many antibiotics. Here, we report the bactericidal action of SkQ1 and dodecyl triphenylphospho-nium (C_12_TPP) against *Rhodococcus fascians* and *Mycobacterium tuberculosis*, pathogens of plants and humans. The mechanism of the bactericidal action is based on the penetration of SkQ1 and C_12_TPP through the cell envelope and the disruption of the bioenergetics of bacteria. One, but probably not the only such mechanism is a decrease in membrane potential, which is important for the implementation of many cellular processes. Thus, neither the presence of MDR pumps, nor the presence of porins, prevents the penetration of SkQ1 and C_12_TPP through the complex cell envelope of *R. fascians* and *M. tuberculosis*.

## 1. Introduction

The penetration of antibiotics through the cell envelope of bacteria is a complex and often unpredictable process. Archetypical Gram-negative bacterium *Escherichia coli* is surrounded by a thin peptidoglycan cell wall and an outer membrane containing lipopolysaccharide, whereas archetypical Gram-positive bacterium *Bacillus subtilis* lacks the outer membrane [1]. Moreover, bacterial cell envelopes differ not only between Gram-positive and Gram-negative bacteria but between different types of Gram-positive bacteria. Typically, a monoderm envelope of Gram-positive bacteria is comprised of a thick peptidoglycan layer of a single cytoplasmic membrane topped by teichoic or lipoteichoic acids, whereas some Gram-positive bacteria, (e.g., *Negativicutes*) have a diderm envelope similar to Gram-negative bacteria [2]. In addition, a fairly large group of Gram-positive bacteria display a complex cell wall composition comprising the mycolyl-arabinogalactan-peptidoglycan (mAGP) complex [3].

The process of a substance penetrating cells does not occur passively along the concentration gradient or electrophoretically due to the difference in electrochemical potentials on the bacterial membrane. This process involves both MDR pumps, which provide the efflux of foreign substances, and porins, which execute their influx [4,5,6]. Thus, the trans-membrane penetration of substances is a multidirectional complex process, of which the precise experimental evaluation is not an easy task per se.

In addition, MDR pumps are often pleiotropic for their substrates. A particular pump may exercise the efflux of many substrates, whereas a single substrate can be expelled by several pumps [5]. In this regard, the mitochondria-targeted antioxidant and antibiotic SkQ1 may serve as an excellent experimental model compound, since under physiological conditions it is output from cells by a single AcrAB-TolC pump [7,8]. Meanwhile, antibiotic resistance provided by deletion mutations in genes for any of the AcrAB-TolC pump proteins parallels the resistance of *B. subtilis* and *Staphylococcus aureus*, prominent representatives of Gram-positive bacteria with monoderm envelopes [7,8,9]. The barrier of the outer membrane is easily penetrated by SkQ1, suggesting either the participation of porins in its transport, or the direct passage of this lipophilic cation through lipid membranes [10].

While penetration through the diderm envelope looks relatively easy, penetration involving the mAGP complex is a more complex process. This specifically concerns the antibiotic resistance of *Mycobacterium tuberculosis* [11]. The permeability of substances for mycobacteria relies on porins, and the diffusion barrier for hydrophilic molecules is 100 to 1000-fold higher than that of *E. coli* [12,13]. The permeability of SkQ1 through the cellular envelope of mycobacteria remains unassessed. For the present work, we selected two pathogenic species from the order *Mycobacteriales*—the human pathogen *M. tuberculosis* and the plant pathogen *Rhodococcus fascians*.

*M. tuberculosis* needs no special introduction: tuberculosis (TB) is one of the oldest diseases known to affect humans and about a quarter of the world’s population is infected with *M. tuberculosis* [14]. According to the WHO, TB is a major death factor in human populations, and the estimated incidence of antibiotic resistant TB varies between 3.4% for primary and 18% for previously treated cases [14,15]. Phytopathogenic bacteria cause many serious plant diseases around the world, although the economic damage for agriculture from bacterial diseases is relatively lower than that from fungi and viruses [16,17]. However, with an annual increase of average summer daily temperatures of 3–4 °C, the prevalence of plant infection grows by 30–50% [18]. *R. fascians* is a phytopathogen which affects a wide range of dicotyledonous and monocotyledonous plants, herbaceous plants, and a few woody species spanning 164 species in 43 plant families. *R. fascians* has been proven to cause proliferation of multiple shoots and stunted growth and proliferation of buds in leaf axils [19,20,21,22]. Whereas *Rhodococcus* isolates lacking virulence genes are beneficial for plant growth, acquisition of plasmids carrying virulence genes is sufficient for the transition of beneficial symbionts into phytopathogens [23]. Streptomycin and oxyguinoline sulfate are commonly used for greenhouse treatments, which reduces losses from *R. fascians* more than five-fold. However, antibiotic therapy causes a delay in rooting and cuttings and raises concerns about elevation of antibiotic resistance in the environment [19,24]. Thus, the introduction of new antibiotics to which phytopathogenic bacteria are sensitive would be beneficial for agriculture [8].

**Figure 1:**
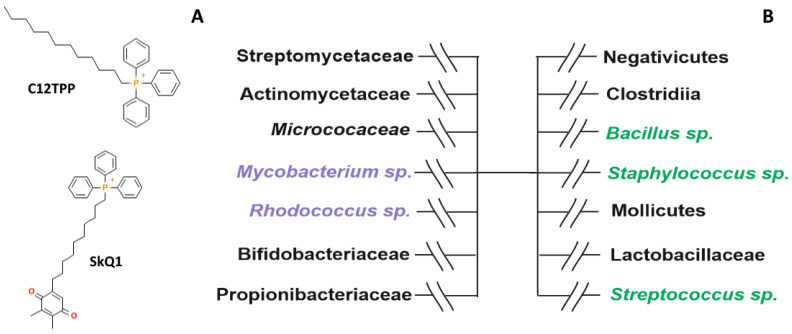
Chemical structures of C_12_TPP and SkQ1 (**A**); Major groups of Gram-positive bacteria (**B**): high G+C (**left**) and low G+C (**right**). Green—SkQ1-tested representatives, black—not tested, purple—tested in this study.

Mitochondria-targeted antioxidants (MTA), which include SkQ1 and dodecyl triphenyl phosphonium (C_12_TPP), are known to alleviate mitochondrial oxidative damage associated with a variety of diseases (Figure 1A). MTA are widely used in experiments for evaluating the impact of mitochondria on different pathological processes involving oxidative stress [25,26,27]. The mechanism of MTA antibacterial action is still debatable, largely due to limited numbers of the strains tested. This specifically concerns Gram-positive bacteria with complex, mycolic acids-based cell walls [7,9,28].

Here we demonstrate that prototype pathogenic, high G+C, Gram-positive bacteria (Figure 1B), *R. fascians* and *M. tuberculosis*, are sensitive to MTA and provide evidence that the antibacterial mechanism relies upon a decrease in membrane potential. This suggests that complex cell walls of Gram-positive bacteria are permeable for MTA under physiological conditions.

## 2. Results

### 2.1. Minimum Inhibitory Concentrations (MIC) for Gram-Positive Bacteria Are Similar

We compared MICs for pathogens under study with those for *B. subtilis*, *S aureus*, and non-pathogenic *M. smegmatis*. All microbes displayed identical MICs = 1–2 μM (Table 1). Earlier, we observed similar results for laboratory *E. coli* strains bearing identical sequences of pump proteins responsible for drug resistance [29].

**Figure 2:**
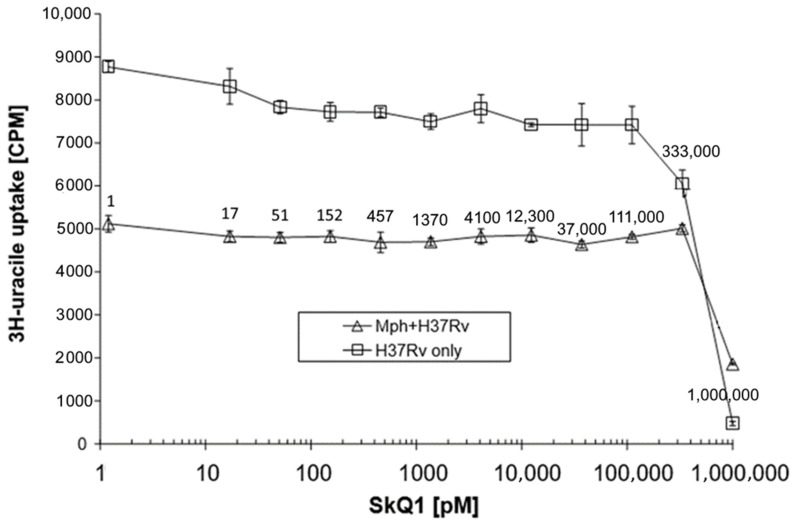
Effect of SkQ1 on the vulnerability of mycobacteria in monoculture and within macrophages (Mph).

Next, we demonstrated that the inhibitory effect of SkQ1 molecules on *M. tuberculosis* cells required identical concentrations, irrespective of whether mycobacteria were cultured in vitro or within peritoneal mouse macrophages (Figure 2). Thus, our results provide evidence that SkQ1 is effective against various actinobacteria, including human and plant pathogens. It remains to be determined whether other members of the MTB complex (*M. bovis*, *M. africanum*, *M. microti*, *M. pinnipedii*, and *M. caprae*) are also sensitive to SkQ1, which is highly likely since all Gram-positive bacteria studied so far, including *Mycobacteriales*, were sensitive to SkQ1.

### 2.2. MTA Have a Bactericidal Effect on Gram-Positive Bacteria

**Figure 3:**
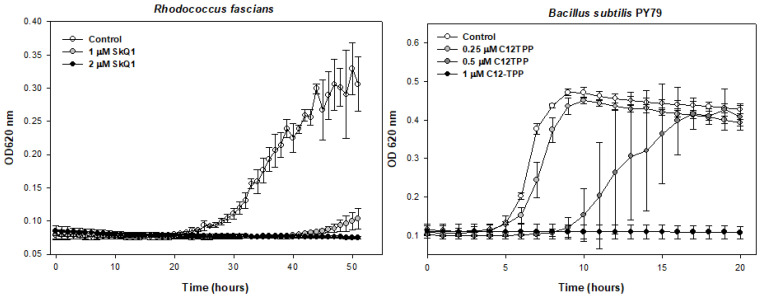
Effect of SkQ1 on the growth of *R. fascians* and C_12_TPP on the growth of *B. subtilis*.

It is necessary to pay attention to the following two facts: (1) there were enough nutrients in the medium so that, at sub-MIC concentrations, the cells reached the maximum possible optical density; (2) when cells were left with added MTA for several days, no growth of bacteria in the medium was observed (Figure 3). This suggests that when the MIC reaches 2 µM, *R. fascians* cells do not show any delayed growth, but most likely die.

To confirm that the action of SkQ1 is bactericidal, we decided to conduct an experiment with rapidly growing B. subtilis cells. A 96-well plate was filled with medium containing 2 μM SkQ1. To six wells, we added 50 µL of cells (2.5 × 10^6^ cells per mL, for the final cell number of 5 × 10^5^ per mL). The 96-well plate was placed in a thermostat at 30 °C. The following day, 50 µL of cells (2.5 × 10^6^ cells per mL) were added to the next six wells. This process was repeated for a total of seven cell additions to the wells with the SkQ1-containing medium that was incubated in the plate.

**Figure 4:**
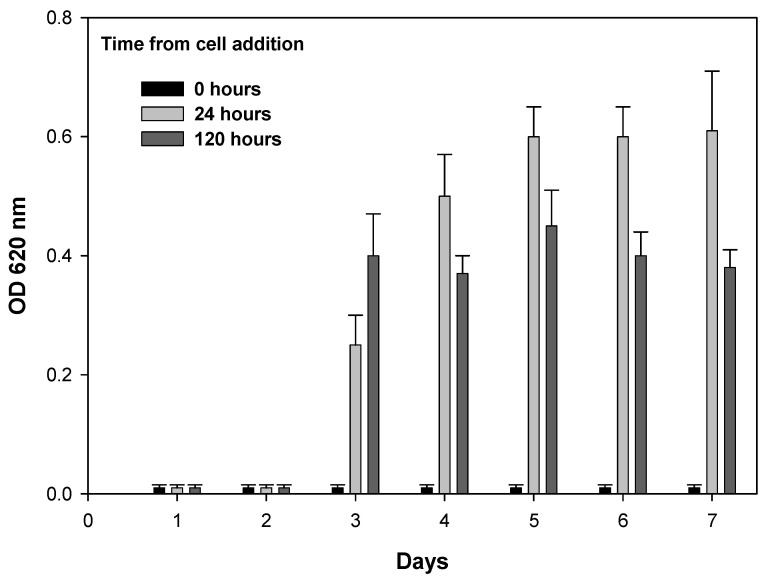
Degradation of SkQ1 in the growth medium. Cells were added to the medium containing 2 μM SkQ1 and incubated for 5 days. OD 620 was measured at the moment when cells were added, after 24 h, and after 5 days. The day on which cells were added to the well is indicated on the *x*-axis. The data points represent mean ± SD of six points in three experiments.

The optical density of the plate was measured every day by using a Thermo Scientific Multiskan FC plate reader. The results are shown in Figure 4. When cells were added in the first two days, no growth was observed, while the growth of the bacterial culture could be detected as early as the third day, which indicates the possible binding/degradation of SkQ1 in the medium at 30 °C. At the same time, for wells with cells added in the first two days, no growth was observed at all even after 12 days of incubation, which apparently indicates the bactericidal effect of SkQ1. The lack of growth was confirmed also by the lack of CFU after inoculating the Petri dishes, which can be considered as the minimum bactericidal concentration. Lower values of optical density after 5 days of incubation are apparently associated with the death of the culture and subsequent necrotrophic growth. Of note, bactericidal activity of SkQ1 is temporary and likely would not cause long-term negative effects on ecosystems and agriculture.

### 2.3. Effect of SkQ1 and C_12_TPP Is Based on Membrane Depolarization

**Figure 5:**
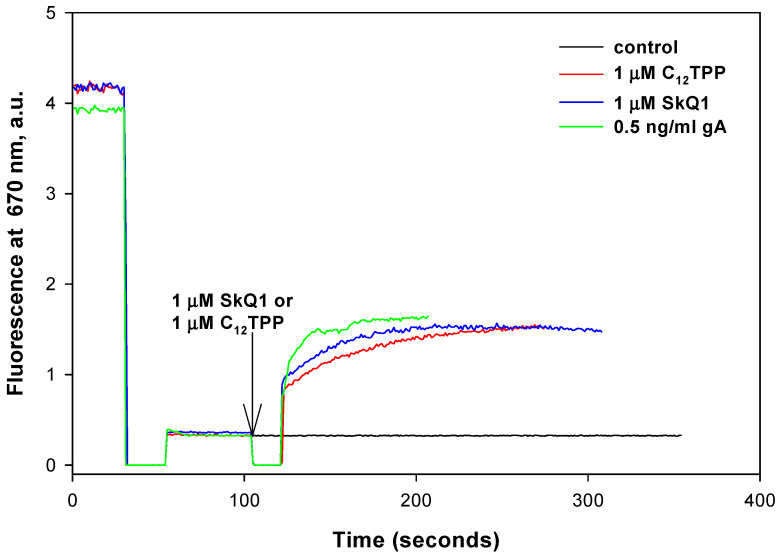
Effect of SkQ1 and C_12_TPP on the membrane potential in *R. fascians*. Changes in the membrane potential were monitored by measuring fluorescence of DiS-C3-(5) (10 µM) in DiS buffer. Gramicidin A (gA) concentration was 0.5 ng/mL.

Further, we first measured the membrane potential of *R. fascians* and examined the effect that triphenylphosphomium derivatives exhibit on it (Figure 5).

Potential-dependent accumulation of the cationic dye DiS-C3-(5) is known to cause its aggregation and lead to fluorescence quenching, therefore the membrane potential of *R. fascians* can be estimated from the fluorescence of the potential-sensitive dye DiS-C3-(5), similar to the case of *B. subtilis*. As shown in Figure 5, 1 μM concentrations of SkQ1 or C_12_TPP caused a rapid decrease in the membrane potential of *R. fascians* in the minute time scale (SkQ1 and C_12_TPP addition marked by an arrow) to the level observed with the channel-forming antibiotic gramicidin A known to deplete the bacterial membrane potential. Thus, all results for *R. fascians* match with those for *B. subtilis* [9].

## 3. Discussion

Despite the fact that Gram-positive bacteria have a fairly significant arsenal of pumps that can effectively remove phosphonium derivatives from cells, the observed ejection of SkQ1 and C_12_TPP by these pumps is ineffective. As we can see, the Bmr pump from *B. subtilis* is not able to have a significant effect on both compounds, similar to the NorA pump from *S. aureus*, although both of these pumps are involved in the removal of phosphonium derivatives [30,31]. Just as in Gram-negative bacteria [32,33,34], the EmrE pump in *E. coli* does not significantly affect the pumping of C_12_TPP and SkQ1. *Mycobacteria* also have their own Mmr pump [35], which removes tetraphenylphosphonium, but is also unable to pump out SkQ1 and C_12_TPP. Thus, despite the great diversity of MDR pumps in Gram-positive bacteria, especially in the order of *Mycobacteriales* [36], the results of experiments indicate that MDR pumps are not involved in the removal of SkQ1 and C_12_TPP from bacterial cells of *R. fascians* and *M. tuberculosis*.

It is well known that the hydrophobic tail enhances permeability through the macrophage cell membrane, and thus improves the bactericidal activity of the corresponding substance against intracellular mycobacteria [37]. In the case of SkQ1 and C_12_TPP, we observe efficient penetration through the Gram-positive bacteria cell envelope, which is expected for mitochondria-targeted compounds [38,39], due to some similarity between bacteria and mitochondria [40,41]. It should be noted that the difference between bacteria and mitochondria is also because of the presence of MDR pumps in bacteria. If the pumps cannot recognize or remove the substance from the cell, then the result of penetration into both the bacterium and mitochondria would look similar.

**Figure 6:**
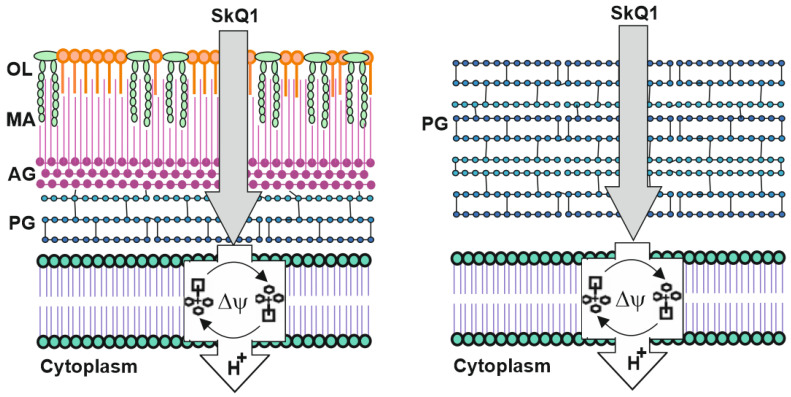
Schematic representation of the mechanism of action of SkQ1 on high G+C Gram-positive bacteria (**left**) and low G+C Gram-positive bacteria (**right**). PG—peptidoglycan, AG—arabinogalactan, MA—mycolic acids, OL—outer lipids. H+—proton leakage, Δψ—membrane potential.

Therefore, the effect of TPP derivatives on pathogens *R. fascians* and *M. tuberculosis* showed that the cell wall is not an obstacle to the penetration of antibiotics based on TPP (Figure 6). As shown earlier [7], even the outer membrane of Gram-negative bacteria was not a sufficient barrier to TPP derivatives, and especially SkQ1. By removing the protein components of the AcrAB-TolC pump, the only SkQ1 pump, the resistance of *E. coli* deletion mutants became comparable to that of Gram-positive bacteria such as *S. aureus*, *B. subtilis*, and *Streptococcus mutans* [7,9,28]. The data obtained in this work on *R. fascians*, *M. tuberculosis*, and previously obtained on *M. smegmatis* indicate that neither high G+C Gram-positive bacteria, nor low G+C Gram-positive bacteria appear to be resistant to TPP antibiotics, supporting the concept that resistance to TPP derivatives depends solely on the composition of MDR pumps in resistant bacteria [42]. For Gram-positive bacteria with a complex cell wall, such as *Negativicutes* [2], resistance will presumably also depend on the presence of MDR pumps capable of pumping out TPP-based lipophilic compounds.

Resistance due to the permeability of the cell envelope is determined not only by the presence of MDR pumps, but also by the presence of porins mediating transport to the bacterial cell membrane. Attention should be drawn to the fact that porins are present in Gram-positive bacteria of the *Mycobacteriales* order, as well as in diderm bacteria. The absence of the AcrAB-TolC pump in *E. coli* leads to resistance comparable to that of *B. subtilis*, which may indicate that porins, along with penetration through membranes beyond SkQ1 and C_12_ TPP lipophilicity, may be the main gates that determine their penetration through the cell envelope [7,8,9]. On the other hand, mycolic acid cell walls contain porins different from those of Gram-negative bacteria, but the cell envelope was also found to be permeable to SkQ1 and C_12_TPP.

Thus, free penetration in the absence of MDR pumps through various porins of both Gram-negative and Gram-positive bacteria indicates that either SkQ1 and C_12_TPP pass directly without the participation of porins, or their structure is such that they can use both groups of porins. Undoubtedly, the refinement of the model of their penetration mechanism will require additional experimental confirmation.

Although the mechanism of action of TPP derivatives on the bacterial cell remains uncertain, it is obvious that the description of it as a non-specific detergent action appears incorrect. Neither the outer membrane of Gram-negative bacteria, nor the mycolic acid cell wall of Gram-positive bacteria affect the MICs of TPP-based lipophilic antibiotics. At the same time, it should be noted that at concentrations several orders of magnitude higher than the MIC, the detergent effect cannot be excluded, but at concentrations comparable to the MIC, a decrease in the membrane potential is apparently observed due to the protonophoric action of TPP derivatives. Although the protonophoric action plays an important role, there is no direct evidence yet that it is the only one responsible for the bactericidal action. Moreover, the presence of a more pronounced protonophoric activity does not infer the presence of a stronger antibacterial activity [43]. Thus, a decrease in membrane potential is one of the mechanisms of action, while not necessarily the main one.

At the same time, it must be remembered that antibiotics that affect the bioenergetics of mycobacteria can have an antagonistic effect on other bactericidal antibiotics. For example, the bactericidal antibiotic bedaquiline, which blocks the proton pump for ATP synthase of mycobacteria [44], may antagonize other bactericidal antibiotics, such as isoniazid and moxifloxacin, at subinhibitory concentrations [45]. This opens up possibilities for creating promising fusion molecules, e.g., where the fusion of the conventional antibiotic chloramphenicol with a CnTPP molecule can lead to both a decrease in the membrane potential and an inhibition of protein synthesis [46,47].

In addition to bactericidal capacity, a potential antibiotic should be non-toxic for human cells. Although triphenylphosphonium derivatives are generally considered as toxic substances, their actual toxicity is often exaggerated. In our recent work [48], it was shown that triphenylphosphonium derivatives are effective against bacterial cells and, to some extent, toxic to single-cell suspensions of human cells; however, they are unable to cause significant damage in cell monolayers or tissues. Mycobacteria reside within macrophages and are able to inhibit phagosome–lysosome fusion which provides a niche with close to neutral pH values. This allows not only survival during antibiotic therapy but even mycobacterial multiplication.

SkQ1 has a multidirectional effect on bacterial cells. Like other triphenylphosphonium derivatives, it inhibits metabolism [48], which is consistent with the data we showed earlier [7,9]. TPP derivatives prevent cell adhesion to the surface, which can affect the course of pathogenesis and prevent the formation of biofilms. In addition, TPP derivatives prevent cell adhesion to the surface, which can affect the course of pathogenesis and prevent biofilm formation. In addition to the negative effect on bacteria, SkQ1 has a positive effect on mammals, and at concentrations toxic to bacteria increases the healthspan and lifespan of rodents [49].

Summing up, we can conclude that mitochondria-targeted antioxidants are extremely effective against bacteria with a mycolic acid-containing cell wall. They reduce the membrane potential, causing disturbances in bacterial bioenergetics, which indicates the great potential of these compounds as an effective tool for combating Gram-positive pathogens.

## 4. Materials and Methods

### 4.1. Materials

SkQ1 was synthesized, as described in ref [10]. C_12_TPP was purchased from Alfa Aesar (Karlsruhe, Germany). Components of bacterial media such as peptone and yeast extract were purchased from Becton, Dickinson and Co., (Franklin Lakes, NJ, USA), agar and NaCl were purchased from AppliChem (Darmstadt, Germany), tryptone was purchased from Helicon Company (Moscow, Russia), and Mueller–Hinton medium was purchased from HiMedia Laboratories (Mumbai, India). Other reagents were purchased from Sigma-Aldrich (St. Louis, MO, USA).

### 4.2. Bacterial Strains and Routine Growth Conditions

Standard strain *Rhodococcus fascians* (Tilford) Goodfellow 1984 strain VKM Ac-1462T was provided by the All-Russian Collection of Microorganisms (VKM). Tryptone yeast extract (TYE) medium (tryptone 10 g, glucose 5 g, yeast extract 5 g, NaCl 5 g, H_2_O 1L) was used as recommended by VKM.

Standard laboratory strain *Bacillus subtilis* subs. *subtilis* Cohn 1872, strain BR151 (trpC2 lys-3 metB10) was used. *Bacillus subtilis* strain PY79 was provided by E.A. Kubareva (Belozersky Institute of Physico-Chemical Biology, Moscow State University, Moscow, Russia). *Staphyllococcus aureus* Rosenbach 1884 (entry #144) and *Mycobacterium smegmatis* (Trevisan 1889) Lehmann and Neumann 1899 (entry #377) were from the Microorganisms Collection of the Moscow State University.

*Mycobacterium tuberculosis* (Zopf 1883) Lehmann and Neumann 1896, strain H37Rv, sub-strain Pasteur, was from the collection of the Central Institute for Tuberculosis (Moscow, Russia). Dubos broth base (BD) medium supplemented with 0.5% fatty acid-poor BSA (Calbiochem) was used.

The bacterial cells were grown at 28 °C, 30 °C, or 37 °C in LB, MH, BD, or TYE medium at the 140-rpm shaking frequency.

### 4.3. MIC Determination

The growth suppression assay was performed by inoculating 200 µL of bacterial cultures (~5 × 10^5^ cells/mL) into 96-well plates (Eppendorf AG, Hamburg, Germany). The compounds were diluted in a 96-well microtiter plate to final concentrations ranging from 0.05 to 10 µM SkQ1 or C_12_TPP in a 250-mL aliquot of the total volume. The bacteria were allowed to grow for 18, 52, or 72 h at 30 °C.

MICs, the lowest concentrations that completely inhibited the bacterial growth, were determined by Mueller–Hinton or TYE broth microdilution, as recommended by CLSI, using in-house-prepared panels [50].

Bacterial growth of *R. fascians* was observed visually alongside the CFU and OD measurements [7]. Optical densities at 620 nm were obtained by using a Thermo Scientific Multiskan FC plate reader with an incubator (Thermo Fisher Scientific, Waltham, MA, USA). Experiments were carried out in triplicate.

The *M. tuberculosis* strain was maintained and prepared for in vitro studies exactly as previously described [51]. Briefly, 50 µL from a thawed 10^8^ CFU/mL aliquot was added to 30 mL of BD media and incubated for 2 weeks at 37 °C. The resulting suspension was washed two times at 3000 g, 20 min, 4 °C, with Ca- and Mg-free PBS containing 0.2 mM EDTA and 0.025% Tween 80. It was then resuspended in PBS with 0.025% Tween 80 and filtered through a 5 μm-pore-size filter (Millipore) to remove clumps. To estimate the CFU content in the filtrate, 20 μL from each 5-fold serial dilution was plated onto BD Dubos agar medium, and the total number of micro-colonies within the spot that was visible on the air-dried agar was calculated under an inverted microscope (200× magnification) after being cultured for 3 days at 37 °C.

Mycobacterium MIC evaluation was performed as described in [52]. Briefly, each inoculum containing 500 × 10^3^ H37Rv mycobacteria was cultured in antibiotic-free RPMI-1640 medium supplemented with 10 mM HEPES buffer, 2% heat-inactivated fetal calf serum (FCS), and 2 mM L-glutamine in a well of the fiat-bottom 96-well plate for 1 days at 37 °C, 5% CO_2_. SkQ1 from a stock solution was serially 3-fold diluted in supplemented RPMI-1640 medium and added to mycobacterial cultures at gradually declining concentrations. Plates were additionally incubated for 3 days. A total of 2 μCi of [^3^H]-uracil (Isotope, St. Petersburg, Russia) per well was added for the last 18 h. The [^3^H]-uracil uptake by mycobacteria was measured by using a Wallac 1409 liquid scintillation counter (Turku, Finland). MIC was defined as the lowest concentration of a compound providing 99% inhibition of [^3^H]-uracil incorporation, compared to the drug-free control wells. All samples were tested twice in triplicates.

MIC evaluation for intra-macrophage mycobacteria: peptone-elicited peritoneal mouse macrophages were obtained and purified as described earlier in detail [49]. MIC was determined in accordance with [52]. A total of 5 × 10^4^ peritoneal macrophages were plated in wells of flat-bottom 96-well plates (Costar-Corning) in antibiotic-free RPMI-1640 medium supplemented with 10 mM HEPES buffer, 5% heat-inactivated fetal calf serum (FCS), and 2 mM L-glutamine. Cells were allowed to adhere for 2 h at 37 °C and 5% CO_2_ and, thereafter, were infected with *M. tuberculosis* H37Rv at the MOI = (1:1). SkQ1 was diluted in supplemented RPMI-1640 and added at indicated concentrations after the first 18 h of co-culture. After 48 h of incubation, 1 mCi/well [^3^H] uracil (Isotop, St Petersburg, Russia) was added for the last 18 h of culture. The cultures were terminated by freezing the plates at −80 °C and harvesting on the fiberglass filters (Scatron, Norway) was performed after thawing the contents. The results ([^3^H] uracil uptake by mycobacteria, cpm) were measured in triplicate in a liquid scintillation counter (Wallac, Finland).

### 4.4. Measurement of R. fascians Membrane Potential

The membrane potential in *R. fascians* was estimated by measuring the fluorescence of the potential-dependent probe DiS-C3-(5) according to [53] with modifications. *R. fascians* were seeded into fresh TYE medium, followed by growth for 52 h until reaching the optical density 0.7 at 600 nm. Then, the bacteria were diluted 20-fold in a DiS buffer containing 100 mM KCl, 10 mM Tris, pH 7.4. The fluorescence was measured at 690 nm (excitation at 622 nm) by using a Fluorat-02-Panorama fluorimeter.

### 4.5. Estimation of Bactericidal Activity of SkQ1

A total of 200 μL of medium containing 2 μM SkQ1 was added to a 96-well plate. On the first day, inoculums (the number of cells was equivalent to 5 × 10^5^ cells/mL) of *B. subtilis* cells were added to 6 wells, and left to incubate for 24 h at 30 °C. The experiment was carried out for 5 days, repeating the procedure. Optical density was measured, daily, at 620 nm by using a Thermo Scientific Multiskan FC plate reader.

## Figures and Tables

**Table 1:** Susceptibility (MIC) of bacterial strains to the TPP derivatives.

	Strain	SkQ1 MIC (µM)	C_12_TPP MIC (µM)	Source
*Rhodococcus fascians*	VKM Ac-1462^T^	1–2	1	This study
*Mycobacterium tuberculosis*	H37Rv	1–2	ND	This study
*Bacillus subtilis*	BR151	1	0.5–1	[7,9]
*Bacillus subtilis*	PY79	1	1	This study
*Staphylococcus aureus*	MSU collection #144	1	1	[7], this study
*Mycobacterium smegmatis* ^1^	MSU collection #377	0.5–1	0.5–1	[7], this study

^1^ Previously referred to as *Mycobacterium* sp.

## Data Availability

Not applicable.
